# Supplementary material for: Risk averse reproduction numbers improve resurgence detection
Source: PLoS Comput Biol. 2023 Jul 20;19(7):e1011332. doi: 10.1371/journal.pcbi.1011332 (PMC10393178; doi:10.1371/journal.pcbi.1011332)
Supplement: Attachment — Submitted filename: Responses D-22-01819.pdf [file pcbi.1011332.s002.pdf]

Responses to Reviewers: PCOMPBIOL-D-22-01819

We thank the reviewers and editors for their constructive and insightful comments. We list our responses to each point and corresponding changes to the main text below. We also include both clean and tracked-change versions of the new text to highlight our revisions. References given here are in a Bibliography at the end of this document.

Editors:

In particular, referee 2 provides a number of comments on ways to strengthen the case for the effectiveness of the proposed quantity, and referee 1 raises relevant points for discussion, including the inter-connectedness of sub-populations.

We strengthen the case for the risk-averse reproduction number *E* in two main ways. First, we provide mathematical arguments on sub-population inter-connectedness and how it may be accommodated within our framework in a new Methods subsection. Second, we include new empirical analyses of COVID-19 outbreaks in Norway, New Zealand, Illinois and New York in the USA as well as for local authorities in the UK and analyse the times that *E* and *R* signal resurgence (new Results subsections). These hopefully clarify and bolster the practicality and usefulness of our proposed metrics. Further, we provide clarifying text in line with the other points raised by reviewer 2. These responses are developed in full in the subsequent sections and new figures are included in the main text as well as the S1 Appendix. Additionally, we expand the Introduction and Discussion to address remaining points of reviewer 1, including more nuanced and descriptive text.

Reviewer #1:

The manuscript by Drs. Parag and Obolski deals with the analysis of weighting schemes aimed at improving the performance of the effective reproduction number (R) as a means of detecting resurgent epidemic dynamics. The authors' basic idea is that current approaches to estimate R from surveillance data tend to overplay the role of larger subpopulations, possibly leading to delays in identifying important signals from smaller subpopulations where transmission rebounds might actually start. To circumvent this limitation, the authors apply some information-theoretic principles from experimental design theory to present two alternative weighting schemes, one based on D-optimality (which, for the problem at hand, reduces to equal weighting for the effective reproduction numbers of the various subpopulations, independently of the active infections circulating therein) and the other based on E-optimality (in which the reproduction number of each subpopulation is weighted according to the fraction of the total reproduction number sum attributable to that subpopulation), respectively. Using numerical experiments of increasing complexity (from a toy model to a realistic application based on actual data), they propose that E-optimal reproduction numbers can produce an effective bias-variance trade-off.

The topic of the manuscript is undoubtedly interesting, as R is widely used in decision-making, despite its known limitations---one of which in fact being the underlying homogeneity assumption often made when estimating it from data. The analysis is well conducted and quite effectively explained, although the nitty-gritty of the methodology might be better understood

by readers with some prior exposure to information theory. The manuscript is sufficiently well written and organized. Specific comments follow.

Many thanks for the positive and detailed appraisal.

Main comments

- 2nd page, 2nd paragraph: The homogeneity assumption is often done when trying to reconstruct R from data; this is not always the case, instead, when the estimation of R is based on other approaches, such as compartmental models calibrated with surveillance data. This family of models certainly has its own share of problems and limitations but can in principle accommodate heterogeneous populations with a computational cost that is typically negligible compared to that of individual-based approaches. A comment on this might help put the initial discussion on the benefits and limitations of R in a broader perspective.

This is a great point. We include a new paragraph in the Introduction on compartmental models discussing how they can account for heterogeneous transmission characteristics e.g., by including multiple infectious classes. While these do not possess the computational expense of network or agent-based models (which incorporate heterogeneities explicitly) they do require knowledge of increasing numbers of parameters and distributions as their complexity (and descriptive power) increase. We distinguish these from the renewal and autoregressive models that are commonly used for real-time estimation and forecasting, and which underlie our investigation. Hopefully this better contextualises our results.

- 4th page, 2nd paragraph: I think that neglecting inter-group infections may perhaps be the most important limitation of this work, which expands (and likely improves) over previous homogeneous approaches by introducing a principled weighting of the different subpopulations but does (understandably) little to account for the epidemiological interrelationships among them---or with the "outside world". The redistribution of information described in equation (4) is said to be able to account for "migrations" (a term that I suggest replacing with something less spatially and temporally connotated). However, the cited reference [28] makes explicit use of mobility fluxes among different subpopulations, the mapping of which onto the modelling framework presented in this manuscript does not appear completely straightforward. Moreover, the other reference cited in this context [21] makes an explicit distinction between local and imported cases, but it is not immediately clear how to introduce this feature in the alpha factors of equation (4) either. I believe that providing more information on how the proposed method can accommodate (or be extended to accommodate) such mixing or import dynamics would represent an important contribution toward a wider applicability of the method.

Thanks for highlighting this issue. We agree that accounting for inter-connectedness among the groups is the natural next step towards a richer and perhaps more accurate description of heterogeneous spreading process. We replace the term migrations with introductions in the text and provide a detailed explanation in the Methods on how **Eq (4)** can be adapted to model these introduction or interconnection dynamics. We repeat this explanation below. Our main argument centres on how the interconnections among groups, from the perspective of the information available for inferring reproduction numbers, causes a redistribution that satisfies our alpha constraint. This conforms to our framework but comes with the difficulty of having to

estimate the fluxes among groups using assumptions as in [1] ([28] in the original text). If instead data on which infections are local versus introduced are available, then as in [2] ([21] in the original text), we can generalise the total infectiousness terms in our renewal framework (i.e., the Λ_j) to include that data without changing the results of our optimal designs.

Specifically, let $\rho_{x \to j}$ be the probability of an infection being introduced into group j from group x as in [1], with $\rho_{x \to x}$ as the probability of remaining in the source group. For p groups $I_j \sim \mathbf{Pois}\left(\sum_{x=1}^{p} \rho_{x \to j} \Lambda_x R_x\right)$. As a result, I_j contains information about the R_x. If we assume that introductions have the infectiousness of their source group and that we know the source group of the introductions, then the informative component of I_j is $I_j | R_x \sim \mathbf{Pois}\left(\rho_{x \to j} \Lambda_x R_x\right)$ (via a Bernoulli thinning). The Fisher information from I_j about R_x is $\rho_{x \to j} \Lambda_x R_x^{-1}$. We may collect the information about R_x available from all the infection data by summing these components as $\sum_{j=1}^{p} I_j | R_x \sim \mathbf{Pois}(\Lambda_x R_x)$ because of the infinite divisibility of the Poisson formulation and as $\sum_{j=1}^{p} \rho_{x \to j} = 1$. Consequently, the total Fisher information about R_x from all the infections is $\mathbf{FI}[R_x] = \Lambda_x R_x^{-1}$. This yields the same D- and E-optimal designs as in the main text and hence our results are valid under this formulation.

This formulation assumes that the introductions have the infectiousness of their source group. This assumption holds for example when heterogeneity is mostly due to regional pathogenic variants, with variants forming groups that have different reproduction numbers. Other forms of heterogeneity are also accommodated, such as if groups demarcate areas with different socioeconomic bands and members maintain their original (source) reproduction numbers as they interact. The alternative, where introductions have the reproduction number of their sink group leads to $I_j \sim \mathbf{Pois}\left(\sum_{x=1}^{p} \rho_{x \to j} \Lambda_j R_j\right)$. If we let $\sum_{x=1}^{p} \rho_{x \to j} = a_j$, then we also obtain a diagonal FI matrix but with terms $\mathbf{FI}[R_x] = a_x \Lambda_x R_x^{-1}$. This will fit our framework if we constrain the sum of all the $a_x \Lambda_x = \alpha_x$ to still be Λ. Sink-based reproduction numbers may occur, for example, if groups define areas with different population densities so imports into an area will acquire the distinct reproduction number of that area .

Note that for all these cases we require knowledge of the introductions or their $\rho_{x \to j}$ values. If the latter are unknown, an alternate approach is to simply treat introductions from each group as input data in accordance with [2]. This means the total infectiousness is redefined as $\Lambda_j = \sum_{u=1}^{s-1} w(u)\left(I_j(t-u) + M_j(t-u)\right)$ with I_j as the local infections of the group and M_j counting introductions into that group. As Λ_j is treated as known we do not depart the framework of the main text and we recover our optimal designs (albeit with this different Λ_j). This convergence of results largely emerges from the fact that, as long as we can ascertain the source and sink of infections, we can correctly assign them to the reproduction numbers that they inform about and construct a diagonal FI matrix. Other models of inter-connections which instead propose inter-group reproduction numbers (e.g., [3]) do not directly fit our framework. These models can be over-parametrised and would admit non-diagonal FI matrices.

- page 12, fig 3 caption: "but overamplifies noise" is there a quantitative way to qualify this statement? I think it could indeed help explore some of the trade-offs involved in the selection of a weighting scheme for a consensus reproduction number.

We replace “overamplifies” with magnifies. This terminology refers to the max R_j metric which always reports the peak of a fluctuating process and hence maximises sensitivity to stochastic changes in reproduction numbers. We quantified this noise effect in **Fig 2** of the main text where the R_1 metric (equivalent there to max R_j) showed the largest standard deviation by a substantial margin. Our case study of Israel (**Fig 5**), together with a new analysis of COVID-19 in Norway (**Fig A** of the S1 Appendix), emphasise this point and show the relatively noisier nature of max R_j as compared to *R*, *D* and *E*.

Other comments
- page 3, 2nd paragraph: "it optimises... estimate uncertainty" unclear, please explain

We clarify in the text that uncertainty relates explicitly to the variance of the R_j estimates and hence the optimisation is precisely the E-optimal design by definition (as variance is inversely proportional to Fisher information and we optimise the Fisher information of every group).

- page 8, 3rd paragraph: "Both D and E... each group" unclear, please explain

We expand on this to highlight that the active infections in each group compose the Fisher information of their reproduction number estimates. This then factors into the uncertainty in *D* and *E* and so both metrics implicitly include the impact of those active infections. In contrast, *R* directly weights the groups by those active infections, which can cause a loss of sensitivity to groups that have key resurgence signals (which often occur at low active infection counts). We also rephrased this sentence in the text to clarify the issue to the readers.

- page 12, fig 3 caption: "do not signal control" quite vague, please rephrase

We restate that they do not indicate subcritical spread (i.e., values below 1).

- page 12, 1st paragraph: "every group... is controlled", "being driven towards elimination” is quite vague too, please rephrase (groups are likely neither controlled nor eliminated, disease transmission is)

Thanks for spotting this ambiguity. We now make explicit that it is disease transmission within those groups that becomes controlled or is driven so that infections become eliminated.

- page 14, 3rd paragraph: "These are reasonable" what: hypotheses/assumptions/...?

We clarify that this relates to our assuming that case reporting is stable and that serial intervals are good proxies for generation times.

- page 18, equation (7): are the age-of-infection indexes correct in the right-hand side expression, or are they switched?

Thanks for checking – they are correct but can be switched without error since the convolution sums to the same value as $\sum_{s=1}^{t-1} w(s)I(t-s)$.

- page 20, first paragraph: algorithm [49], to derive -> algorithm [49] to derive

Thanks, corrected now.

Reviewer #2:

In this study, the authors propose an alternative measure to the standard effective reproduction number (R), the risk averse reproduction number (E). The new measure aims at addressing some issues with the standard definition of R such as the low sensitivity to local resurges which might lead to a delayed detection of outbreak resurgence and therefore have important ramifications for policy responses.

Even though the proposed metric is interesting, the authors should try to refine/revise the manuscript to make a more compelling case of its usefulness. In the following I will provide a few comments to address this concern.

Thanks for the constructive critique and the helpful suggestions. Hopefully the usefulness of *E* is now better motivated and demonstrated.

First, from the title and abstract of this paper, the stated goal of the authors is to promote the new measure E. However, in the remainder of the paper and especially in sections “D and E optimal reproduction numbers and their properties”, “Risk averse reproduction numbers are more representative of key dynamics”, and “Empirical application to COVID-19 across 20 cities in Israel” the authors actually focus on two “risk averse reproduction numbers”. This takes the reader’s focus away from their proposed main metric (E) which is the one discussed at the beginning and end of the paper. For this reason, I would recommend the authors to either move the discussion about D in the supporting information or to broaden their statements at the beginning and end of the paper to promote (more in general) “optimal-design/risk averse reproduction numbers” (i.e. both D and E).

Thanks, our intention was simply to present *D* as another optimal solution but one which does not have the risk-averse properties we seek (which *E* does). In a sense *D* serves as a null model to contrast to, together with the max function. We have modified the text to better clarify the purpose of *D* and to emphasise that *E* is our main contribution. Our new **Figs 6-7**, which examine multiple empirical datasets, excludes *D* to clarify we are interested in *E*.

Second, the “synthetic examples” showcase situations in which E might be better at predicting resurgence than R, however it is not clear whether those simulations come from an “organic model” or rather from independent simulations of local outbreaks which are then simply assumed to be part of the same (global) geography. Let me clarify this point. If one of the main arguments brought forward is that E is better than R at detecting a local resurgence that might become a global resurgence, then the authors should provide synthetic simulations in which - for example - they use a metapopulation model showing that a local resurgence in a context of connected subpopulation will transform into a global resurgence because of the delayed R-dynamics (vs E-dynamics) that will prevent a timely policy response.

We agree with the reviewer’s comment and address it in several ways. First, we added new mathematical analysis of connectivity between groups. We show above in our response to the

second comment of reviewer 1 and in a new Methods subsection that, under some common models of connectivity, our *E* statistic still solves the E-optimal design problem and therefore has the same risk-averse properties as in the independent case. The renewal models in these interconnected cases differ (either possessing modified Λ_j constructions or factors that define the likelihoods of infections moving across or among groups) but the functional relationship between the Fisher information about each R_j available from the infection data remains largely consistent. Even when alternate approaches to assessing connectivity that sit outside our framework are used, we would still expect *E* to be more risk-averse than *R* and to generally provide earlier warnings about the possibility of local resurgences leading to global ones.

We illustrate this latter point in both a model and simulation agnostic way using mathematical arguments that we include below and in a new section of the S1 Appendix. If we hold the R_j of our groups constant and project forwards in time (a standard assumption when forecasting infectious diseases [8,9]) then we would find that the rank (in magnitude) of the Λ_j becomes more correlated with the rank of the R_j as time increases. This means that the group with the largest local R_j tends eventually to also have the largest circulating infection count. Hence, we gain some predictive insight if we can meaningfully assign weights to match the ranks of the R_j while accounting for local estimate uncertainty. *E* does this directly, setting weights to match the fractional rank of R_j. Of course, the R_j will change with time but *E* will then update its resurgence forecast to reflect these changes. However, *E* is more than just predictive as it also ensures that risky groups (i.e., those with larger R_j that have higher chances of rapid spread and lower chances of infections naturally fading out) are emphasised relative to *R*.

This follows as it is a Lehmer mean, $\left(\sum_{j=1}^{p} R_j^{q}\right)\left(\sum_{j=1}^{p} R_j^{q-1}\right)^{-1}$ with $q = 2$. Lehmer means are used in signal processing as envelope detectors (i.e., to detect the peaks in a waveform) and interpolate between the arithmetic mean $q = 1$ and the maximum of its inputs $q \to \infty$, which also relate to *D* and the max R_j statistics respectively. Therefore, *E* will always have a risk-averse property without being overly sensitive like the larger q means. This property holds for any inputs but the added benefit within our renewal model description is that *E* also handles uncertainties in the R_j estimates well because it is the unique solution to the E-optimal design problem. By comparison, *R* is a weighted arithmetic mean and has no guarantees on either its optimality or its ability to detect risky groups. Moreover, as we showed in **Fig 1**, it tends to underestimate uncertainty (in contrast to max R_j, which overestimates uncertainty). Taken together this means that *R* will be delayed and confident in its delayed signalling. We add some of the above explanations into the Discussion as well as the S1 Appendix.

Second, we take the reviewer's later suggestion and demonstrate the value of *E* using multiple further case studies that involve empirical data, instead of focussing on synthetic approaches (see our responses below, which include new analyses of 5 COVID-19 datasets). These data show local resurgences that grow into global ones (seen from their summed incidence curves). We chose not to perform metapopulation simulations as they require a number of assumptions (e.g., parametrising the matrix of probabilities of infections moving among connected regions) and because we wanted to reflect how reproduction numbers are commonly used practically. During the COVID-19 pandemic *R* was estimated both countrywide and sometimes also at the

level of local districts, authorities or cities composing that country (these form our groups). As connectivity probabilities and fluxes were frequently unavailable, local estimates were treated independently (e.g., in [4–7]). Resulting values for the R_j then relate to *R* as in our formulation (**Eqs. (2)-(3)**). Our simulations (**Figs 2-4**), which are independent and summed, mimic this usage, showing how we might infer transmissibility from local to global levels in practice.

However, the propagation of the epidemic across the groups is important. The performance of *E* relative to *R* on the multiple empirical case studies (new **Figs 6-7** and also **Figs A-C** of the S1 Appendix) further reinforces that *E* maintains its risk-averse properties during real epidemic propagation scenarios and local-global resurgences. Note that if local epidemics are synchronised as in the UK COVID-19 example (which acts as a null model), *E* and *R* converge. We expand on this performance of *E* relative to *R* in more detail in the later responses below. In combination with our earlier mathematical arguments, we hope we have demonstrated the improved resurgence prediction *E*, both realistically and generally.

Third, related to the point above, it would be useful if the authors were to show the “policy response” in action using R vs E (for example in synthetic simulations). In the example of the metapopulation model above, this would entail running simulations in which - on the one hand - the timing of the policy interventions is driven by changes in R and - on the other hand - a scenario in which - instead - the timing is dictated by changes in E. This would provide a way to quantify the policy impact of using E vs R. Furthermore, this could also help identifying cases in which even E might be moving “too slowly” to prevent local resurgences to become global problems (i.e. are there cases in which the surges are so rapid that both R and E, especially when accounting for delayed reporting of surveillance data, fail to provide enough lead times for policy makers to react?).

While we appreciate this point and why it would be insightful, modelling policy response is outside the scope of this work and a genuine analysis would require its own paper. Specifically, policy response is usually controlled by numerous measures of which the reproduction number may not be the most important (e.g., political and economic metrics may be better predictors). Further, if we do assume policy is a simple function of whether *R* or *E* goes above or below 1, we would need to have some notion of the cost of acting to meaningfully model response and whether it is worth responding earlier or not. Otherwise, because acting via *E* or *R* will always reduce the R_j we would be guaranteed to ‘flatten the curve’ more quickly using *E*.

Various studies which have examined economic and optimal costs to intervention timing [10–12] also provide strong evidence that acting earlier is almost always more beneficial. We base our suggestions of the benefits of *E* on this research and on its mathematical properties. *E* by dint of the optimal design problem it solves and its formulation as a contraharmonic or Lehmer mean (see response above for mathematical details) will be likely to signal resurgence earlier and will be more conservative than *R* when supporting intervention relaxation. These traits are all apparent and consistently visible across our 6 empirical datasets (see responses below), which we analyse in lieu of performing intensive modelling.

The question of when changes are too rapid for responses via signals from *E* or *R* is equivalent to asking about what makes an epidemic uncontrollable. This largely depends on the quality of surveillance data (e.g., delays and case under-ascertainment limit our ability to detect and

respond to detected growth in the epidemic [13,14]) and the characteristics of the pathogen (e.g., pre-symptomatic spread and long incubation periods make it difficult to control spread [13,15,16]). These are fundamental to the epidemic and surveillance system in question and not a property of either *E* or *R* specifically. Consequently, as these factors are external (they would also limit the inherent accuracy of early-warning systems [17,18]), we do not detail these effects. However, we now include text in the Discussion sketching some of these arguments.

Fourth, in the section "Empirical application to COVID-19 across 20 cities in Israel" the authors show that using E might have helped in deciding to start the booster campaign earlier in time. Could the authors quantify this? For example, by comparing the dates in which E estimates were above 1 vs when R estimates were above 1 (considering also the role played by the uncertainty of such estimates).

We now include explicit dates or time differences over which $R > 1$ and $E > 1$ for Israel and provide plots of these differences for all 6 empirical datasets in the new **Figs 6-7**. We compare the times when the 2.5% quantile of the Bayesian posterior estimates of *R* and *E* cross 1 (i.e., when the lower 95% credible interval crosses 1) as well as the times when $\mathbf{P}(R > 1) > 0.95$ and $\mathbf{P}(E > 1) > 0.95$. Both measures have been previously used to quantify crossing times in infectious disease with account of uncertainty [7,19]. We comment in the Results about the implications of these earlier times both in the context of Israel and more generally. Note that in the UK regions empirical dataset there is no crossing time difference between *R* and *E*. This serves as a null model and occurs because local incidence curves are largely synchronised.

Last, we note that these measures offer a conservative view of resurgence and that another approach could analyse when mean estimates of the reproduction numbers cross 1 or when the same resurgence probabilities cross 0.5. In these cases, the lags are even larger. We include estimates of these for Israel in detail in the S1 Appendix. In short, we find that for Israel the conservative metrics showed $E > 1$ and $R > 1$ on the 21 and 23 June 2021 whereas the mean metrics indicated these crossing on the 8 and 20 June, respectively. For some of the studies such as New Zealand, New York and Illinois there was more than 2 weeks difference between when $E > 1$ and $R > 1$ under the conservative measures.

Fifth, in the Discussion, the authors identify one of the possible Achille's heel in promoting the use of E vs R: the risk averse reproduction number requires infection time and incidence data (or proxies of them) at the local resolution. The authors do not seem to make a strong case in addressing this weakness rather than a general concession to the fact that "real-time decision-making depends on quality of data". As an addition to that statement, it would be useful if the authors could show more than one real world example in which they applied their proposed metric. In other words, can the authors provide additional case studies (like the one provided for Israel) showing that indeed – for example in the case of the "variants waves" of COVID-19 – this approach could have been used in different countries to fine tune the timing of the booster campaigns (or of other interventions)?

Thanks for this suggestion. We now analyse COVID-19 data from a total of 6 diverse datasets covering large and small scales of transmission in the new **Figs 6-7**. Our new results show, in line with the theory, that *E* signals resurgence more quickly than *R* and would recommend the earlier enaction of interventions. We also include **Fig B** of the S1 Appendix on Norway showing

that *E* would indicate an earlier initiation of the booster campaign there as well. Additionally, *E* is slower to recommend relaxation of any existing interventions as the epidemic becomes controlled. These two properties are why we describe *E* as a risk-averse reproduction number. We include text on these effects in the Results and S1 Appendix (also see response above).

COVID-19 data is available at the highest resolution of any epidemic or pandemic to date and it is expected that the surveillance of infectious diseases will only improve. Consequently, the requirement of local resolution data is unlikely to be limiting. If such data are unavailable not only can *E* not be computed, but also no local level analysis at all is possible i.e., if we cannot compute the R_j that *E* depends on there is little we can learn. In fact, because *E* derives from renewal models it only necessitates incidence and generation time information. This contrasts both compartmental and individual-based modelling approaches, which need knowledge of additional rates of recovery, latent periods, contact patterns and other distributions. Despite this, we maintain that all metrics in infectious diseases strongly depend on surveillance data quality. This is a fundamental bottleneck that cannot be solved at the level of models. We add some of these points to the Discussion of the manuscript.

In other words, one of the main weaknesses I see with this manuscript in its current form is that it does not make a strong compelling case for real world applications of the proposed E measure, even though that seems to be the biggest selling point of this novel metric. Therefore, I would recommend the authors to improve either the “synthetic simulations” part of the manuscript by using more realistic scenarios or/and by adding additional case studies besides the ones they already consider.

Thanks for this useful critique, which has helped improve the manuscript. As described above, we focussed on realistic scenarios instead of metapopulation modelling to avoid making too many assumptions and to show exactly how *E* can be applied in practice. Hence, we included 5 additional empirical examples covering COVID-19 transmission across countries, cities and districts in the UK, USA (New York and Illinois), New Zealand and Norway. These are diverse datasets subject to heterogeneities in contact patterns, behaviour, sociodemographic factors and interventions yet in all scenarios where those heterogeneities matter to transmission, we find *E* signals upcoming resurgences faster than *R*. In the UK dataset, where groups appear homogeneous in their incidence curves, we find *R* and *E* show equivalence performance with no false positive signalling. Last, we include detailed mathematical arguments in the Methods and S1 Appendix (also explained in the responses above and in comment 2 of reviewer 1) to show why we expect our framework to generally improve upon *R* in real world applications.

Bibliography

1. Bhatia S, Lassmann B, Cohn E, Desai AN, Carrion M, Kraemer MUG, et al. Using digital surveillance tools for near real-time mapping of the risk of infectious disease spread. npj Digital Med. 2021;4: 73. doi:10.1038/s41746-021-00442-3
2. Roberts MG, Nishiura H. Early estimation of the reproduction number in the presence of imported cases: pandemic influenza H1N1-2009 in New Zealand. PLoS One. 2011;6: e17835. doi:10.1371/journal.pone.0017835
3. Glass K, Mercer GN, Nishiura H, McBryde ES, Becker NG. Estimating reproduction numbers for adults and children from case data. J R Soc Interface. 2011;8: 1248–1259. doi:10.1098/rsif.2010.0679

4. Abbott S, Hellewell J, Thompson RN, Sherratt K, Gibbs HP, Bosse NI, et al. Estimating the time-varying reproduction number of SARS-CoV-2 using national and subnational case counts. Wellcome Open Res. 2020;5: 112. doi:10.12688/wellcomeopenres.16006.2
5. COVID-19 Re [Internet]. [cited 1 Dec 2020]. Available: https://ibz-shiny.ethz.ch/covid-19-re/
6. Bhatia S, Cori A, Parag K, Others. Short-term forecasts of COVID-19 deaths in multiple countries.
7. Stolerman LM, Clemente L, Poirier C, Parag KV, Majumder A, Masyn S, et al. Using digital traces to build prospective and real-time county-level early warning systems to anticipate COVID-19 outbreaks in the United States. Sci Adv. 2023;9: eabq0199. doi:10.1126/sciadv.abq0199
8. Nouvellet P, Cori A, Garske T, Blake IM, Dorigatti I, Hinsley W, et al. A simple approach to measure transmissibility and forecast incidence. Epidemics. 2018;22: 29–35. doi:10.1016/j.epidem.2017.02.012
9. Funk S, Camacho A, Kucharski AJ, Lowe R, Eggo RM, Edmunds WJ. Assessing the performance of real-time epidemic forecasts: A case study of Ebola in the Western Area region of Sierra Leone, 2014-15. PLoS Comput Biol. 2019;15: e1006785. doi:10.1371/journal.pcbi.1006785
10. Morris DH, Rossine FW, Plotkin JB, Levin SA. Optimal, near-optimal, and robust epidemic control. Commun Phys. 2021;4: 78. doi:10.1038/s42005-021-00570-y
11. Morgan ALK, Woolhouse MEJ, Medley GF, van Bunnik BAD. Optimizing time-limited non-pharmaceutical interventions for COVID-19 outbreak control. Philos Trans R Soc Lond B, Biol Sci. 2021;376: 20200282. doi:10.1098/rstb.2020.0282
12. Haw DJ, Forchini G, Doohan P, Christen P, Pianella M, Johnson R, et al. Optimizing social and economic activity while containing SARS-CoV-2 transmission using DAEDALUS. Nat Comput Sci. 2022;2: 223–233. doi:10.1038/s43588-022-00233-0
13. Casella F. Can the COVID-19 Epidemic Be Controlled on the Basis of Daily Test Reports? IEEE Control Syst Lett. 2021;5: 1079–1084. doi:10.1109/LCSYS.2020.3009912
14. Parag KV, Donnelly CA, Zarebski AE. Quantifying the information in noisy epidemic curves. Nat Comput Sci. 2022;2: 584–594. doi:10.1038/s43588-022-00313-1
15. Fraser C, Riley S, Anderson RM, Ferguson NM. Factors that make an infectious disease outbreak controllable. Proc Natl Acad Sci USA. 2004;101: 6146–6151. doi:10.1073/pnas.0307506101
16. Kahn R, Peak CM, Fernández-Gracia J, Hill A, Jambai A, Ganda L, et al. Incubation periods impact the spatial predictability of cholera and Ebola outbreaks in Sierra Leone. Proc Natl Acad Sci USA. 2020;117: 5067–5073. doi:10.1073/pnas.1913052117
17. Dablander F, Heesterbeek H, Borsboom D, Drake JM. Overlapping timescales obscure early warning signals of the second COVID-19 wave. Proc Biol Sci. 2022;289: 20211809. doi:10.1098/rspb.2021.1809
18. Parag KV, Donnelly CA. Fundamental limits on inferring epidemic resurgence in real time using effective reproduction numbers. PLoS Comput Biol. 2022;18: e1010004. doi:10.1371/journal.pcbi.1010004
19. Nishiura H, Miyamatsu Y, Mizumoto K. Objective determination of end of MERS outbreak, South Korea. Emerg Infect Dis. 2016;22: 146–8.
