# Supplementary material for: Risk averse reproduction numbers improve resurgence detection
Source: PLoS Comput Biol. 2023 Jul 20;19(7):e1011332. doi: 10.1371/journal.pcbi.1011332 (PMC10393178; doi:10.1371/journal.pcbi.1011332)
Supplement: Attachment — Submitted filename: Responses D-22-01819_second.docx [file pcbi.1011332.s003.docx]

**Responses to Reviewers: PCOMPBIOL-D-22-01819**

We thank all reviewers and editors for their constructive and helpful comments throughout. We list our responses to the remaining points of reviewer 2 below. We also include both clean and tracked-change versions of the new text to highlight our revisions.

**Editors:**

Based on the reviews, we are likely to accept this manuscript for publication, providing that you modify the manuscript to revise and improve the section on ABMs vs. network and compartmental models as indicated by Reviewer 2.

Many thanks for the positive viewpoint. We have now revised the section on ABMs, networks and compartmental models to include more nuance, detail and references that summarise more recent advances in those models. Hopefully, the description is now better balanced and reflective of the characteristics of all three modelling paradigms. For more information see our responses below and the tracked changes version of the main text.

**Reviewer #2:**

The authors answered and clarified all main concerns of this reviewer.

Many thanks for the positive comments. We are happy that the revisions proved satisfactory.

As for the new additions to the paper, I would like to kindly ask the authors to improve/revise the part of the manuscript in which they discuss agent-based models (ABMs) vs network models vs compartmental models.

"This is a great point. We include a new paragraph in the Introduction on compartmental models discussing how they can account for heterogeneous transmission characteristics e.g., by including multiple infectious classes. While these do not possess the computational expense of network or agent-based models (which incorporate heterogeneities explicitly) they do require knowledge of increasing numbers of parameters and distributions as their complexity (and descriptive power) increase. We distinguish these from the renewal and autoregressive models that are commonly used for real-time estimation and forecasting, and which underlie our investigation. Hopefully this better contextualises our results."

While this new section certainly provides some additional clarity, it is somehow still a bit confusing.

We hope that our updated treatment of this section as described below and visible from the tracked changes of the main text provides a better and more accurate view of these models. For convenience we also include the relevant paragraphs at the end of these comments.

For example, distinguishing between ABM and network models is quite an arbitrary distinction as individual-based network models are ABMs; and all agent-based models can be also seen through the lens of being network models. Furthermore, the citations provided by the authors seem quite outdated. A lot of work has been done in this space in the last 10/15 years.

We have included updated references and no longer claim substantive distinctions between ABMs and network models. We group their characteristics and discussion, but we do note that network models may perhaps be classified by an emphasis on getting the underlying structure of contacts (not just at individual but also at community levels) accurately specified.

If it is certainly true that agent-based models require high-resolution data (to run/parametrize the model, not necessarily to fit them), recent years have shown how the availability of - for example - large-scale mobility datasets has enabled the creation of very detailed ABM models (see for example recent works by Alberto Aleta). Furthermore, it has been shown that ABMs can be used to create stratified contact matrices (e.g. by age, see Mistry, D., et al. Inferring high-resolution human mixing patterns for disease modeling. Nat Commun 12, 323, 2021) which can in turn be used to introduce heterogeneous mixing in more traditional compartmental modeling approaches (including metapopulation models).

Thanks, we have included these key points in the revised section. We have not been too detailed, however, as the manuscript only aims to sketch and provide pointers about these other important approaches to heterogeneity .

In addition, network models do not necessarily need detailed contact tracing data as contact networks can be approximated either by using contact matrices (built via ABM sims or survey data) or by using high-resolution mobility data (e.g. SafeGraph, Cuebiq, etc..) which can be used to map individual behaviors (e.g. visitation to different POIs) during an average day, therefore allowing for the creation of “probabilistic” contact networks (see A. Aleta works).

We briefly mention that other large-scale datasets can be used and generalise from specifying any dependence on having detailed contact tracing data.

Lastly, also when discussing metapopulation models it would be useful to update the citations as a lot of work has been done in recent years, including a lot of contributions during the recent COVID-19 pandemic (e.g. see recent works by Lauren Meyers’ group, Jeffrey Shaman’s, Alessandro Vespignani’s group, etc..).

We now include the additional and more modern references as suggested.

To put it differently, this new section is a bit “rough around the edges” and it would benefit from another round of edits. Furthermore, I would like to point out that the suggested citations/authors listed above are merely examples, the main point I am trying to put across is that this section still needs some work.

Thanks for all of these suggestions, which we believe have notably improved this section:

The benefits of $R$ mainly stem from two properties: it is easily interpretable as a threshold parameter, and it is easily computable in real time, requiring only routine surveillance data such as epidemic curves of cases [11,12]. However, these must be balanced against its core limitation – $R$ is commonly derived under a well-mixed assumption in which individuals are homogeneous and have equal probabilities of encountering one another. Generalising this assumption to account for the fact that realistic contact rates are heterogenous, leading to assortative and preferential mixing [13], often necessitates some loss in either interpretability or computability [14]. As examples, we discuss agent-based [15,16] network [17,18] and compartmental [19,20] models. These are common and overlapping approaches to including heterogeneity in transmissibility that can possess complementary characteristics.

Agent-based and network models explicitly describe individual-level epidemic dynamics [17]. They incorporate structures and rules that define heterogeneous patterns of connectivity or mixing among individuals or larger groups such as communities. Although the distinction can be arbitrary, network models may focus more on capturing interaction patterns while agent-based models may emphasise individual-level behaviour. Both approaches require extensive high-resolution data to parametrise connectivity and can become computationally intractable [21]. Connectivity, and hence transmission heterogeneity, may be directly fit from contact tracing data or approximated from large-scale mobility and survey datasets [22–24]. Further, these models can allow us to probabilistically infer connectivity by comparing their outputs to observed data, leading to stratified contact matrices that define the mixing levels among groups [24–26]. However, there are questions about the interpretability and even existence of reproduction numbers for agent-based and network models [27–29].

In contrast, compartmental models, which class or group individuals by their epidemiological state (e.g., susceptible or infected) and assume homogeneous transmission in their simplest formulation, have explicitly defined reproduction numbers [19]. Compartmental models can be extended to depict more complex interactions by including additional classes, which may be informed by contact matrices derived from empirical data or from previous agent-based or network models. This allows compartmental approaches to represent heterogeneous transmission without incurring as much computational overhead or intractability [19,20]. Unfortunately, these additional classes also require more epidemiological rate or distribution data, limiting real-time usage and reducing the interpretability of outputs.

Minor comment:

In Fig 6 and 7, it would be useful to have the color legend for the 3 curves also in the main figure (as done in the other figures), rather than explaining the color scheme of the lines only in the figure caption.

We have now included legends in those figures as suggested.
